# Unilateral Compressive Peroneal Neuropathy in Intensive Care Settings During the COVID-19 Pandemic: A Series of Three Cases

**DOI:** 10.7759/cureus.65789

**Published:** 2024-07-30

**Authors:** Kavitha Andiappan, Khin Nyein Yin, Muhamad Faizal Zainudin

**Affiliations:** 1 Department of Rehabilitation Medicine, Hospital Sungai Buloh, Sungai Buloh, MYS; 2 Department of Rehabilitation Medicine, Faculty of Medicine and Health Sciences, Universiti Malaysia Sabah, Kota Kinabalu, MYS; 3 Department of Rehabilitation Medicine, Faculty of Medicine, Universiti Teknologi MARA (Majlis Amanah Rakyat), Sungai Buloh, MYS

**Keywords:** nerve entrapment, patient positioning, peroneal nerve paralysis, peroneal neuropathies, peroneal nerve

## Abstract

Peroneal nerve entrapment, typically associated with behaviors like cross-legged sitting or squatting, can also occur from extended periods of lying down where the lower limbs usually assume a position of hip external rotation and knee flexion. In such positions, the fibular head's prominence can exert sustained pressure on the peroneal nerve. We report three cases of unilateral peroneal neuropathy in intensive care unit (ICU) patients during the coronavirus disease (COVID-19) pandemic, highlighting the possible role of prolonged supine or lateral decubitus positions in the development of this condition. Electrophysiological studies confirmed peroneal nerve palsy in all cases, with two patients achieving full recovery, while the third required a permanent ankle foot orthosis for mobility due to a lack of neurological recovery. The COVID-19 pandemic has challenged ideal nursing care, including in ICU settings, leading to suboptimal nursing care standards and compromised frequent positioning regimes.

## Introduction

Peroneal neuropathy is the third most common focal neuropathy after median and ulnar neuropathies [1]. The anatomical pathway of the peroneal nerve, characterized by its superficial course, predisposes it to various traumatic and pressure-induced injuries [2]. The common peroneal nerve (CPN) is often compressed at the fibula’s bony prominence; the superficial peroneal nerve (SPN) gets entrapped as it exits the lateral compartment of the leg; and the deep peroneal nerve (DPN) can be compromised as it crosses underneath the extensor retinaculum [1].

This condition can arise from mechanical and non-mechanical etiologies, leading to unilateral or bilateral manifestations. Mechanical etiologies encompass traumatic incidents such as knee dislocations and fibula fractures. At the same time, non-traumatic origins might include prolonged leg crossing, repetitive stretching from squatting, positioning during anesthesia, excessive and rapid weight loss, and immobilization with castings or orthoses [1-4]. Risk factors such as pre-operative valgus deformity, flexion contracture, and a higher body mass index are linked with an increased incidence of peroneal palsy post-total knee arthroplasty [1]. Iatrogenic damage may also occur during surgery through direct manipulation of the peroneal nerve [4]. In athletes and dancers, forced ankle inversion and plantar flexion can overstretch the SPN, leading to mononeuropathy [1].

Non-mechanical causes are frequently associated with systemic conditions such as diabetes, Charcot-Marie-tooth disease, polyarteritis nodosa, and thyroid disorders [2,3]. Diabetes is by far the most common non-mechanical cause of peroneal neuropathy, where the deposition of sorbitol causes neural edema, leading to compression [5].

Compressive peroneal neuropathy secondary to prolonged immobility is rarely documented. Yu et al. reported four cases of compressive CPN neuropathy following immobility due to alcohol intoxication [6]. Prone ventilation, practiced as an adjunctive treatment to improve oxygenation during the coronavirus disease (COVID-19) pandemic, was also associated with peroneal neuropathy, as reported by Chang et al. (five cases), Hokkoku et al. (one case), and Taketa et al. (one case) [7-9]. This report details three additional cases of unilateral peroneal neuropathy that occurred in the intensive care unit (ICU) during the COVID-19 pandemic. These cases were identified in an outpatient rehabilitation clinic and were retrospectively analyzed through electronic medical records to determine potential causative factors. We hypothesize that the prolonged supine or lateral decubitus positions during ICU stays have resulted in neuropathy, highlighting potential shortcomings in nursing care amid the pandemic.

## Case presentation

Case A

A 68-year-old male with chronic diabetes, hypertension, and ischemic heart disease was admitted to the ICU for category 5 COVID-19 [10]. He developed complications such as right pulmonary artery embolism, bilateral organizing pneumonia, right pneumomediastinum, right pneumothorax, as well as stage 2 sacral pressure injury. Following his transfer to a general ward two weeks later, a left foot drop was observed, likely developed during the ICU stay. The motor power of the left ankle dorsiflexor was rated 3/5 on the Medical Research Council (MRC) scale, and he exhibited sensory deficits at the first toe webspace. The foot plantar flexor, invertor, and evertor motor power were preserved. Following inpatient rehabilitation, he achieved independent ambulation by using a walking frame and a left ankle foot orthosis before home discharge. He continued to attend regular outpatient therapy sessions and demonstrated progressive improvement. Nerve conduction studies (NCS) performed six months post-admission demonstrated absent compound muscle action potential (CMAP) in the left peroneal-extensor digitorum brevis (EDB) and a smaller CMAP of the left peroneal-tibialis anterior (TA) in comparison to the right side, with reduced amplitude and conduction velocity. Needle electromyography (EMG) of the left TA indicated neuropathic motor unit action potential (MUAP) with a polyphasic pattern, positive sharp waves (PSW), and mild fibrillations suggestive of ongoing denervation activity with reduced recruitment. The MUAP of the left peroneus longus was normal. His clinical profile and electromyographic findings were consistent with left DPN palsy. Fifteen months after the onset, the patient fully recovered from the foot drop and no longer required a walking aid or orthosis for independent ambulation.

Case B

A 60-year-old female, also with underlying chronic diabetes, hypertension, and dyslipidemia, was admitted to the ICU for category 5 COVID-19. She also developed COVID-related complications, namely bilateral subsegmental pulmonary embolism, right-sided pneumothorax, and organizing pneumonia. During the ICU stay, she developed a right foot drop associated with numbness over the dorsum of the foot, but there was no pain. Physical examination revealed right ankle dorsiflexor and evertor motor power of grade 3/5, with sensory loss over the dorsum of the foot. The Tinel sign was positive below the right fibular head. NCS performed eight months after the onset demonstrated absent CMAP in the left peroneal-EDB and reduced conduction velocity of the left peroneal-TA. The findings indicated right CPN neuropathy. Her foot drop completely resolved within 11 months following outpatient rehabilitation and regular neuromuscular electrical stimulation over the tibialis anterior muscle.

Case C

A 50-year-old female with chronic diabetes and dyslipidemia was hospitalized due to pulmonary tuberculosis and melioidosis, which escalated to septic shock and hospital-acquired pneumonia. Her ICU admission was stormy with several complications, including cardiac arrest, upper gastrointestinal bleeding, acute kidney injury, and stage 3 sacral pressure injury. Repeated failures to extubate necessitated a tracheostomy and extended mechanical ventilation. The prolonged ICU stay contributed to generalized weakness, with her motor power documented as 3/5 in the upper limbs and 2/5 in the lower limbs, except the right ankle dorsiflexor, which exhibited no response (0/5). She could ambulate independently at discharge using a wide-based quadripod and an ankle-foot orthosis. The NCS performed six months post-admission demonstrated the absence of bilateral sural sensory, bilateral tibial motor, and right peroneal motor studies. Only small responses were recorded for the left peroneal motor. Given the duration and severity of her condition, she was diagnosed with critical illness polyneuropathy (CIP). During the subsequent months, she showed progressive improvement in motor power, except for the right ankle dorsiflexor and persistent loss of sensation between the first toe webspace. After one year, all motor power returned to normal except for the right dorsiflexor, which remained 0/5. We concluded that the patient had a compressive DPN injury over the right leg. She requires a posterior leaf spring ankle foot orthosis to facilitate ambulation for the long term without needing a walking aid and has successfully returned to her previous job as a nurse.

**Figure 1:**
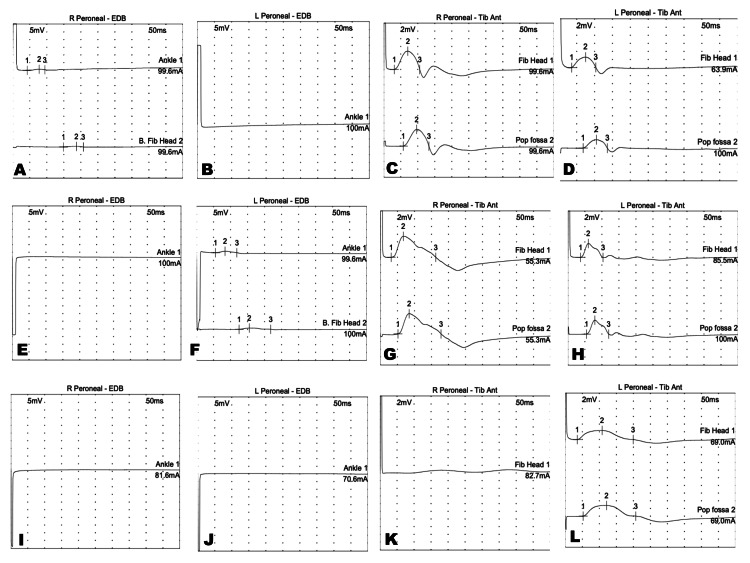
Nerve conduction studies The comparison of the motor NCS waveforms between the right and left peroneal EDB and TA of case A (A-D), case B (E-H), and case C (I-L). EDB: extensor digitorum brevis; NCS: nerve conduction studies; TA: tibialis anterior.

In all three cases, laboratory investigations revealed unremarkable findings that may contribute to the neuropathy, including serum electrolytes and thyroid function tests. The blood sugar levels were reported to be within an acceptable range during the stay. None of the patients reported back pain or radiculopathy symptoms. Figure 1 and Table 1 summarize the NCS findings.

**Table 1:** Results of nerve conduction studies EDB: extensor digitorum brevis; NCS: nerve conduction studies; NR: not recordable; TA: tibialis anterior; ms: millisecond; mV: millivolt, m/s: meter per second.

NCS parameters		Case A		Case B		Case C		Normal value
		Right	Left	Right	Left	Right	Left	
Latency (ms)	EDB	4.19	NR	NR	5.56	NR	NR	≤6.5
	TA	3.1	3.6	5.15	3.1	NR	3.54	≤6.7
Amplitude (mV)	EDB	0.7	NR	NR	1.0	NR	NR	≥2.0
	TA	4.8	2.6	5.3	12.9	NR	2.4	≥3.0
Velocity (m/s)	EDB	33.0	NR	NR	45.0	NR	NR	≥44
	TA	42.0	35.0	40.0	46.0	NR	49.0	≥44

## Discussion

**Figure 2:**
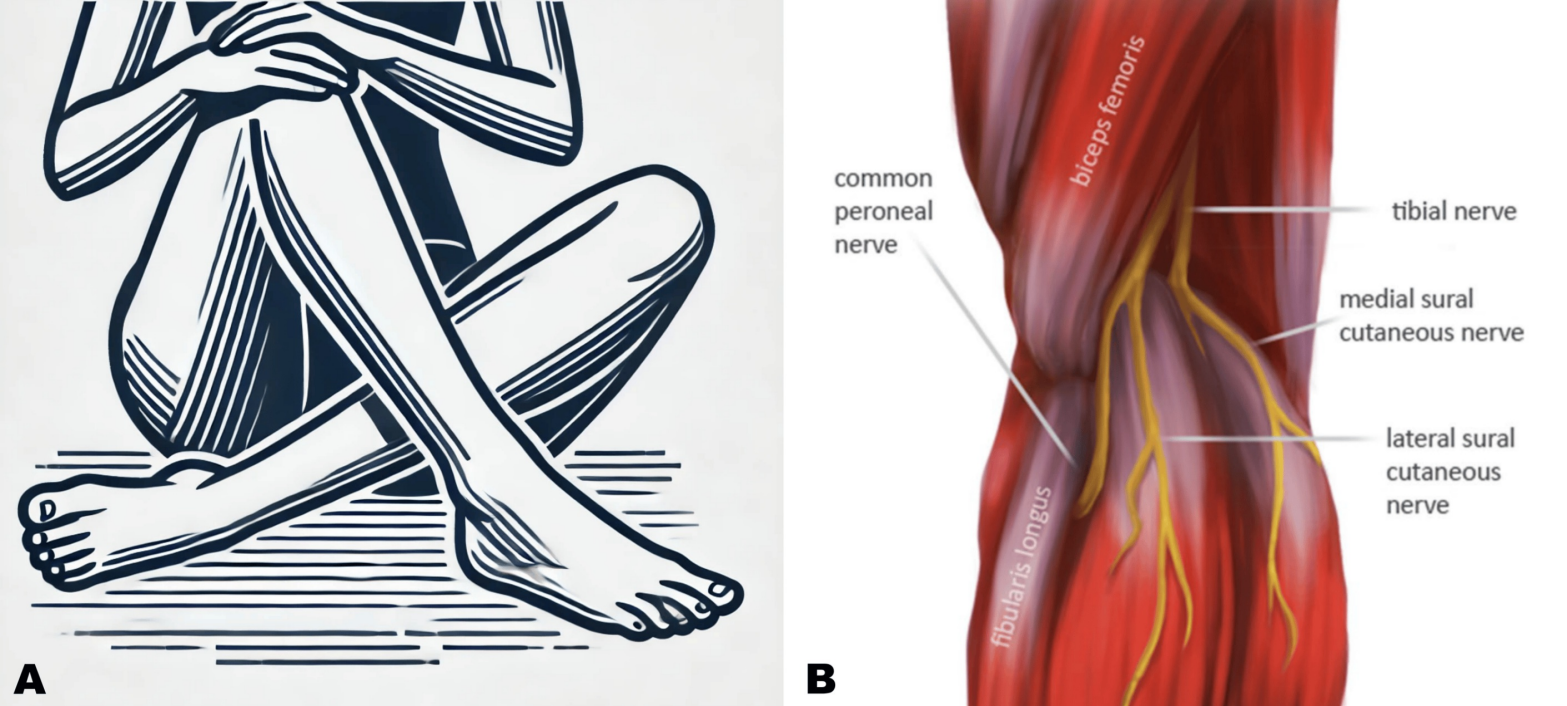
Leg position and the risk of compressive peroneal neuropathy (A) Illustrates cross-legged sitting, commonly practiced in Asian culture; (B) represents the neural pathway of CPN. CPN courses posterolaterally just posterior to the long head of the biceps femoris and moves anteriorly, wrapping around the fibular neck, where it usually gets compressed and passes beneath the lateral compartment of the calf (reproduced with the permission from Capodici et al.: An overview of common peroneal nerve dysfunction and systematic assessment of its relation to falls. Int Orthop. 2022, 46:2757-2763) [2]. CPN: common peroneal nerve.

Posture-induced peroneal neuropathy is a nerve entrapment syndrome presented after maintaining certain positions, such as squatting, kneeling, or habitual leg crossing while seated in Asian culture (Figure 2) [6]. Apart from positional habitudes, peroneal nerve entrapment can also arise from extended periods of lying down [3,6]. Factors such as gravity, the shape of the lower extremity, and muscular imbalance during supine lying contribute to the tendency of the lower limbs to assume a position of hip external rotation and knee flexion [4]. This position, combined with the fibular head’s bony prominence, may exert continuous external pressure on the peroneal nerve, especially in individuals with slim body habitus or excessive weight loss, where the protective fat pad over the fibular head is reduced [3]. Malnutrition is a common comorbid condition with prolonged bed rest, leading to weight loss and potentially contributing to peroneal neuropathy [4]. Additionally, as prolonged bed rest is a risk factor for deep venous thrombosis, pneumatic compression devices (PCDs) are frequently used in the ICU [4]. However, the use of PCDs has been associated with peroneal neuropathy [11].

Various proning protocols have been developed to manage COVID-19-related acute respiratory distress syndrome (ARDS). One commonly used technique is the "Cornish Pasty," which involves positioning the patient in the "swimmer's position" [7]. In this position, the patient's head is turned to one side, with the ipsilateral arm raised and the hip and knee flexed, while the contralateral arm and leg are extended alongside the body. While prone ventilation has shown benefits for patients with COVID-19-related ARDS, including improved oxygenation, this technique, despite instructions for alternating positions every two hours, has been associated with compressive peroneal neuropathy [7-9]. Since prone ventilation was employed in our setting during the pandemic, the observed unilateral peroneal neuropathy in cases A and B could also be attributed to this practice.

Early preventative programs in the ICU can play a crucial role in mitigating the risk of peroneal nerve damage. Using bilateral padded ankle foot orthoses to maintain feet in dorsiflexion and legs in a neutral position, along with frequent repositioning, can effectively minimize peroneal nerve compression risks [4]. This preventative approach is particularly feasible in the Malaysian ICU setting, where a favorable nurse-to-patient ratio of 1:1 is maintained [12]. However, during the COVID-19 pandemic, the ideal nursing care standard was significantly challenged. Healthcare workers (HCWs) faced overwhelming stress, anxiety, burnout, fatigue, or themselves becoming infected, necessitating isolation, leading to compromised patient care [13,14]. In some instances, inexperienced HCWs with insufficient skills and training were launched into the ICUs [13]. Nurses encountered unparalleled stressors due to the overwhelming nature of their responsibilities, bearing witness to widespread suffering and bereavement while enduring prolonged shifts amidst immense strain [15]. Cases of healthcare workers abandoning their posts or refusing to attend to patients suspected of having COVID-19 were not uncommon [13]. These factors may have contributed to inadequate monitoring and prolonged positioning in ICUs, increasing the risk of compressive peroneal nerve injuries.

Neuropathy is a well-documented complication of extended ICU stays that typically manifests as polyneuropathy rather than isolated mononeuropathies [16]. Neurological complications of the COVID-19 infection and its vaccines are not uncommon and include conditions such as stroke, meningitis, and acute transverse myelitis [17,18]. In cases where COVID-19 affects the peripheral nerves, the outcome is predominantly polyneuropathy [19]. Similarly, systemic diseases like diabetes mellitus and thyroid disorders generally lead to multiple nerve impairments, presenting as either polyneuropathy or mononeuritis multiplex [20].

In acute nerve compression, ischemia and intraneural edema can cause conduction blocks, which are often reversible [21]. In most cases of posture-induced peroneal neuropathy, the nerve injury type is neuropraxia by Sunderland’s classification, and remyelination usually occurs over several weeks [6]. However, in chronic compression, the endoneurial inflammatory response triggers the formation of fibroblast-moderated scar tissue that may further exacerbate the compression, leaving long-lasting clinical deficits [21]. Surgical decompression has been shown to improve the prognosis in refractory cases. However, in practice, posture-induced peroneal neuropathy patients tend to undergo passive treatments and wait for treatment by clinicians due to a benign prognosis [6]. It is essential to highlight that significant delays in performing the NCS were observed in all three cases, likely due to service disruptions during the pandemic. These delays could have potentially impacted the prognosis of the patients.

## Conclusions

This case series highlights the critical impact of prolonged ICU stays on the development of unilateral compressive peroneal neuropathy. Extended periods in supine, lateral decubitus, or prone positions can lead to significant nerve compression, resulting in motor and sensory deficits. Two out of three patients fully recovered with conservative management, while one required long-term orthotic support due to persistent neurological impairment. The findings underscore the importance of stringent nursing protocols, frequent repositioning, and improved staffing and training. Frequent repositioning is a key measure for preventing such complications. Early identification and intervention are essential, as posture-induced neuropathies are generally reversible but can lead to prolonged recovery or permanent deficits if not promptly addressed. Enhancing ICU care practices is vital to mitigate nerve injury risks and improve patient outcomes in critical care settings.
